# Nomograms for Predicting Prognosis of Primary Mediastinal Seminoma: A Population-Based Study

**DOI:** 10.1155/2021/9048375

**Published:** 2021-05-19

**Authors:** Weijia Huang, Jingwen Luo, Xianghong Zhou, Yunuo Zhao, Tao Zhang, Xuelei Ma

**Affiliations:** ^1^Department of Biotherapy, West China Hospital and State Key Laboratory of Biotherapy, Sichuan University, Chengdu 610041, China; ^2^Lung Cancer Center, West China Hospital, Sichuan University, Chengdu 610041, China; ^3^West China School of Medicine, Sichuan University, Chengdu 610041, China; ^4^Department of Urology, Institute of Urology and National Clinical Research Center for Geriatrics and Center of Biomedical Big Data, West China Hospital, Sichuan University, Chengdu, Sichuan Province 610041, China

## Abstract

**Objectives:**

Primary mediastinal seminoma (PMS) was an uncommon carcinoma, and the appropriate treatment remained controversial due to the low incidence. We aimed to investigate the demographics and tumor biological characteristics to determine the potential effective treatment and predict the prognosis.

**Methods:**

Patients diagnosed with PMS were selected between 1975 and 2016 from Surveillance, Epidemiology, and End Results (SEER) database. Kaplan–Meier analysis and Cox proportional hazard model were conducted to determine the prognostic factors, and nomograms were employed to visually predict the prognosis. Concordance index (C-index), calibration curve, and receiver operating characteristic (ROC) curve were conducted to validate the prediction model.

**Results:**

A total of 476 patients were included with a median age of 31 years (range, 2–76 years), and a median size of the tumor was 11.6 cm (range, 0.2–24.0 cm). The 5- and 10-year overall survival (OS) rates were 70.4% and 68.4%, respectively. Age, the extent of the primary site, metastatic status, and surgery performance were independent prognostic factors. Not received surgery was considered a poor prognostic factor for OS (HR, 1.86; 95% CI, 1.13–3.03; *P*=0.013). The *C*-index was 0.733 (95% CI, 0.685–0.781) and 0.819 (95% CI, 0.737–0.901) for internal and external validation for predicting OS, respectively. The area under the ROC curve (AUC) was 0.743 (95% CI, 0.681–0.804) for predicting OS (sensitivity, 0.532; specificity, 0.887) in the training cohort.

**Conclusions:**

The nomogram could efficiently predict the survival of patients with PMS. Surgery was the potential effective treatment, and chemotherapy was strongly recommended for patients over 40 years.

## 1. Introduction

Primary mediastinal seminoma (PMS), first reported in 1955, is a comparatively rare subtype of extragonadal germ cell tumor (EGCT) but ranked second of germ cell tumors (GCTs) in the anterior mediastinum, just next to mature teratoma [1–3]. The EGCT histologically contained the same tissues found in the gonadal tissues, while no mass was found in testis among patients with PMS [4, 5]. The common extragonadal positions were mediastinum and retroperitoneum [6], and the incidence of EGCT was around 0.18/100,000 [7]. PMS ranged 37% of all mediastinal GCTs [3], while malignant mediastinal GCTs only accounted for less than 5% of mediastinal tumors [8].

PMS mainly occurred in young males, while fewer in females [8]. The mean age at diagnosis ranged from 25 to 35 years, and there was no increased incidence reported in males but it decreased in females [9]. The 5-year survival of PMS was over 80% [5, 10], while it was around 50% in patients with nonseminomatous germ cell tumors (NSGCTs) [5]. It was found that the tumors, whose primary site was not genital organs, were more relevant to progression or death compared with testicular primary [11]. Age [12–14], metastatic status [6, 15], and operation performance [16] were potential prognostic factors. The appropriate treatment remained controversial, while platinum-based chemotherapy combined with surgery is now the recommended treatment [1, 5, 8, 17].

Due to the low incidence, there would not be a large prospective or single-center retrospective study that showed the prognosis and prognostic factors of PMS among a big crowd [5, 8]. Besides, the individualized treatment strategies and prediction of survival are still not clear. To investigate the demographics and clinical characteristics and determine the effective treatment of PMS, we conducted a population-based cohort based upon the Surveillance, Epidemiology, and End Results(SEER) database.

## 2. Methods

### 2.1. Patient Selection

**Figure 1:**
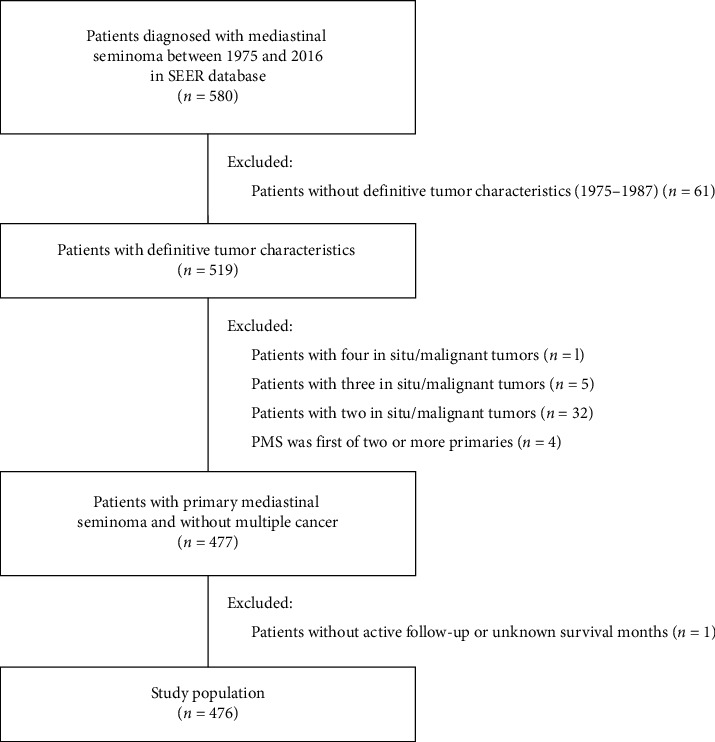
Flowchart describing the inclusion and exclusion criteria in detail and the selection from Surveillance, Epidemiology, and End Results database.

The SEER program has been operated since 1973 by the National Cancer Institute, which consisted of 18 cancer registries and covered approximately 34.6% of the population in the United States. SEER∗ Stat (version 8.3.6; http://www.seer.cancer.gov) was used for our analysis and obtained in February 2020 [18]. Patients diagnosed with mediastinal seminoma between 1975 and 2016 were selected (International Classification of Diseases-10 site code: C38.1-C38.3; International Classification of Disease for Oncology histology code: 9061/3–9062/3, 9064/3) [7]. Since the TNM staging system (the seventh edition) was applied in 2010, the patients involved were identified using the extent of disease (EOD) coding system, which was compatible with the American Joint Committee on Cancer's Manual (the third edition). The inclusion and exclusion criteria are shown in the flowchart in Figure 1. We excluded patients with undefined tumor characteristics (*n* = 61), multiple cancer (*n* = 42), and inactive follow-up (*n* = 1). Thus, a primary cohort was selected, randomly divided into a training group and a validation group with a ratio of 7 : 3.

### 2.2. Demographics and Variables

In the study, demographic variables included sex, age, year of diagnosis, race, and marital status. Tumor-associated variables included EOD, the extent of the primary site, lymph nodes involved, metastatic status, and tumor size. Treatment associated variables included surgery, radiation, and chemotherapy performance. The age was divided into four groups, <20, 20–40, 40–60, and ≥60 years. The year of diagnosis was separated by 2004. The race was divided into three groups: white, black, and other (Asian or Pacific Islander, American Indian/Alaska native, and unknown). The marital status was divided into three groups: single (never married), married, and others (divorced, separated, widowed, and unknown). The tumor characteristics between 1988 and 2003 were based on the EOD-10-digit system, the data between 2004 and 2015 on collaborative stage data collection system, and the data in 2016 on TNM classification (the seventh edition). The EOD was divided into four stages: localized, regional (regional by direct extension only, regional lymph nodes involved only, or regional by both direct extension and lymph node involvement), distant (distant sites/nodes involved), and unknown. The primary site's extent was divided into six phases: invasive tumor confined to the site of origin, localized (not otherwise specified, NOS), invaded to adjacent connective tissue, invaded to adjacent organs/structures, further contiguous extension, and unknown. The tumor size was divided into three groups: <10 cm, ≥10 cm, and unknown. The procedures, radiation, and chemotherapy details were not available in the database. Follow-up time was defined as a period from the time of diagnosis to death or last follow-up.

### 2.3. Statistical Analysis

Categorical variables were described with frequency and proportion. The difference of demographic characteristics, tumor, and treatment characteristics between the training cohort and validation cohort was described by the *χ*^2^ test and log-rank test. The Kaplan–Meier method was conducted to evaluate overall survival (OS) and cancer-specific survival (CSS) in univariate analysis, and survival curves would be employed to show the difference. Cox proportional hazards model was conducted in multivariate analysis to identify independent prognostic factors. A *P* value of less than 0.05 was identified with a statistically significant level.

Nomograms were adopted to visualize the multivariate regression analysis and predict 1-, 5-, and 10-year OS and CSS rates. The concordance index (*C*-index) and calibration curve were conducted to validate the precision of the prediction model. The *C*-index evaluates the discrimination of a survival model, indicating a better prediction when it goes to 1 and a worse forecast when it goes to 0.5 [19]. The calibration curve shows the difference between actual survival and predicted survival, with 1000 resamples of bootstrapping. Afterward, the receiver operating characteristic (ROC) curve was used to validate the concordance of the model, and then the value of the area under curve (AUC) would be given as an assessment indicator. The statistical analyses above were performed by R 3.6.1 (The *R* Foundation for Statistical Computing) and *R* packages (stats, plyr, rms, survival, Hmisc, ROCR).

## 3. Results

### 3.1. Descriptive Characteristics

A total of 476 patients were selected between 1988 and 2016 as a primary cohort, which was then divided into a training cohort (333 patients) and a validation cohort (143 patients). Among the primary cohort, the median age was 31 years (range, 2–76 years), and the follow-up time was 92 months (95% CI, 84–100 months). In total, 462 (97.1%) were male and 387 (81.3%) were white. The demographics and clinical characteristics of the primary cohort, training cohort, and validation cohort are shown in Table 1.

### 3.2. Demographics and Survival

**Figure 2:**
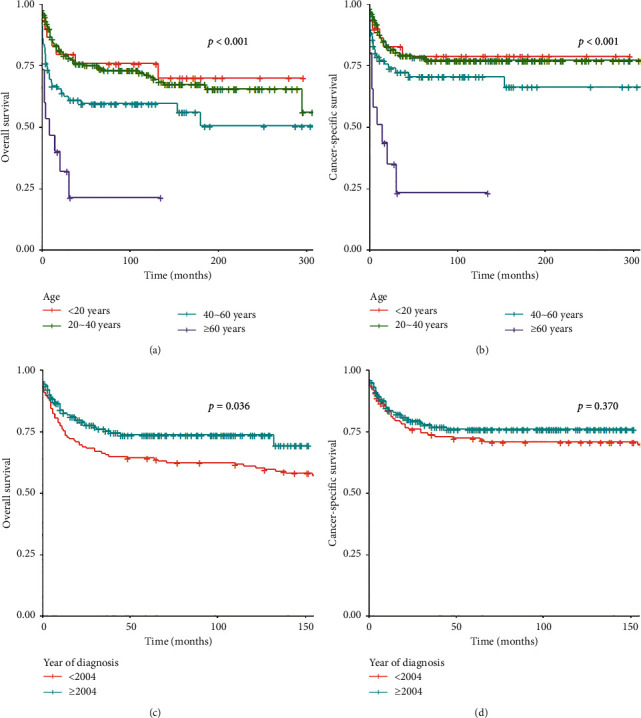
Kaplan–Meier curves comparing survival concerning age ((a) overall survival; (b) cancer-specific survival) and year of diagnosis ((c) overall survival; (d) cancer-specific survival) in the training cohort.

Age, extent of the primary site, metastatic status, and surgery performance were independent prognostic factors (Table 2). Age and year of diagnosis were all significantly correlated with OS (Figure 2), while sex was not associated with OS (*P*=0.445) or CSS (*P*=0.404). Compared to patients with less than 20 years, patients with more than 60 years had a poor prognosis in OS (HR = 5.26; 95% CI, 1.75–15.81; *P*=0.003) and CSS (HR = 7.93; 95% CI, 2.22–28.32; *P*=0.001), and those between 40 and 60 years had an even lower OS (HR = 2.64; 95% CI, 1.09–6.41; *P*=0.032). Moreover, the year of diagnosis was not associated with either CSS (*P*=0.370) or OS (*P*=0.075).

### 3.3. Tumor Characteristics and Survival

The extent of the primary site involved lymph nodes and metastasis were all significantly associated with OS and CSS (*P* < 0.001). The unknown infringed extent of primary tumor was associated with poor OS (HR = 2.56; 95% CI, 1.07–6.13; *P*=0.034) and CSS (HR = 3.36; 95% CI, 1.21–9.31; *P*=0.020). Meanwhile unknown metastasis status was a protective factor for OS (HR = 0.34; 95% CI, 0.13–0.93; *P*=0.035) and CSS (HR = 0.14; 95% CI, 0.03–0.56; *P*=0.006). In the primary cohort, the median size of the tumor was 11.6 cm (range, 0.2–24.0 cm), apart from those unknown cases. However, the tumor size was not associated with OS (*P*=0.246) or CSS (*P*=0.202). Furthermore, no significant difference was observed in the training cohort and validation cohort in baseline variables.

### 3.4. Treatment and Survival

In the primary cohort, 113 (23.7%) patients received surgery, 94 (19.7%) received radiation, and 407 (85.5%) received chemotherapy. The 5-year and 10-year OS rates were 70.4% and 68.4%, respectively. 5-year and 10-year CSS were 74.8% and 74.1%, respectively. Patients without surgery were associated with poor OS (HR = 1.86; 95% CI, 1.13–3.03; *P*=0.013) and CSS (HR = 1.84; 95% CI, 1.02–3.31; *P*=0.041). It was comparable in survival for radiation and chemotherapy. While among patients aged no less than 40, chemotherapy led to a favorable outcome in OS (HR = 0.20; 95% CI, 0.10–0.41; *P* < 0.001) and CSS (HR = 0.20; 95% CI, 0.09–0.49; *P* < 0.001).

### 3.5. Construction and Validation of the Nomogram

**Figure 3:**
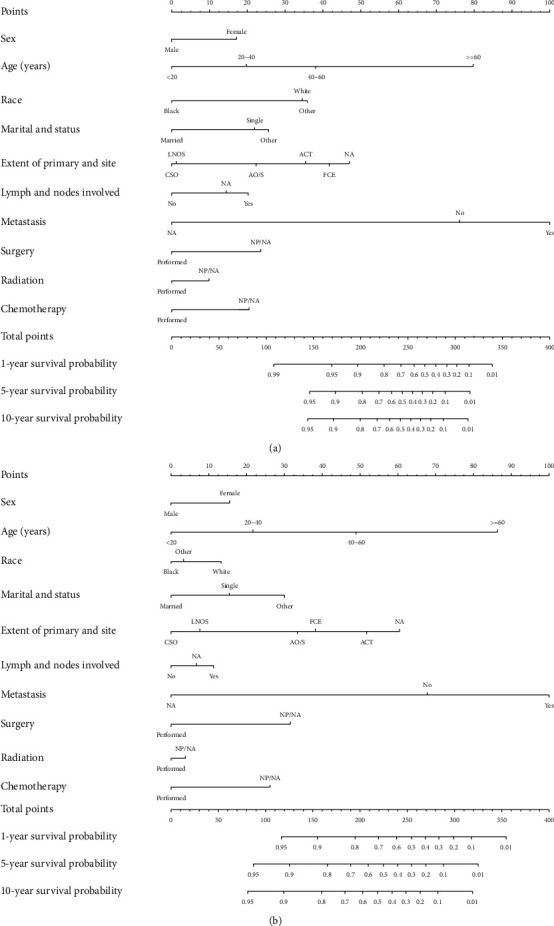
Prediction of 1-, 5-, and 10-year overall survival (a) and cancer-specific survival (b) via nomograms. CSO, confined to the site of origin; LNOS, localized (not of specified); ACT, extend to adjacent connective tissue; AO/S, adjacent organs/structures; FCE, further contiguous extension; NA, unknown; NP/NA, not performed or unknown.

**Figure 4:**
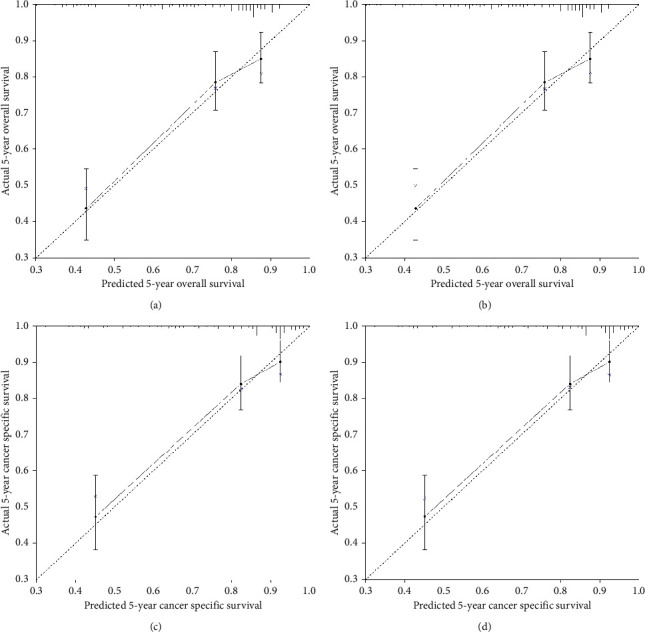
Calibration curves of predicted and actual 5-year overall survival ((a) training cohort; (b) validation cohort) and cancer-specific survival ((c) training cohort; (d) validation cohort) used by internal and external validation in patients with primary mediastinal seminoma.

Nomograms were conducted to predict the 1-, 5-, and 10-year OS and CSS and visualize the prediction models (Figure 3). *C*-index was 0.733 (95% CI, 0.685–0.781) and 0.775 (95% CI, 0.718–0.831) for OS and CSS in the training cohort (internal validation), respectively. *C*-index was 0.819 (95% CI, 0.737–0.901) and 0.860 (95% CI, 0.784–0.936) for OS and CSS in the test cohort (external validation), respectively. The validation of 5-year survival showed a preferable prediction power via calibration curves (Figure 4).

### 3.6. Construction and Validation of ROC Curve

**Figure 5:**
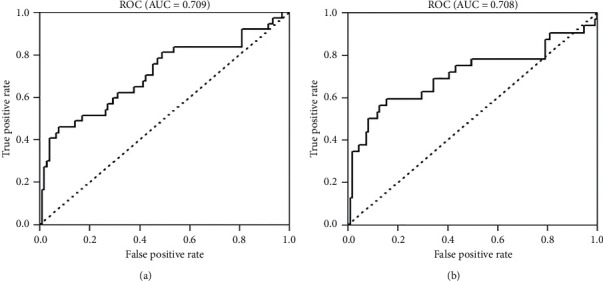
Receiver operating characteristic (ROC) curve for predicting overall survival (a) and cancer-specific survival (b) in the validation cohort.

We also conducted ROC curves to evaluate the prediction ability (Figure 5). In the training cohort, ROC curve showed a moderate performance in OS (AUC, 0.743; 95% CI, 0.681–0.804; sensitivity, 0.532; specificity, 0.887) and CSS (AUC, 0.784; 95% CI, 0.719–0.849; sensitivity, 0.637; specificity, 0.850). The validation cohort also showed a medium prediction power in OS (AUC, 0.709; 95% CI, 0.600–0.817; sensitivity, 0.459; specificity, 0.925) and CSS (AUC, 0.708; 95% CI, 0.584–0.832; sensitivity, 0.594; specificity, 0.847).

## 4. Discussion

PMS is not a common malignancy, and single-center retrospective studies were not likely to conclude practical clinical decisions. We described the demographics and baseline characteristics of PMS between 1988 and 2016 and went over the treatment strategies via the SEER database. Then we identified the prognostic factors via univariate and multivariate analysis and established the prediction model for 1-, 5-, and 10-year OS and CSS showed by nomogram. In our study, the 5-year OS and CSS rates were 70.4% and 74.8%, respectively, which was comparable with previous research. A multicenter retrospective study reviewed the patients between 1975 and 1996 at 11 cancer centers and indicated that the 5-year OS rate was 88%, in which 51 patients were diagnosed [5]. To our knowledge, this is the first most extensive population-based study to describe the clinical characteristics and treatment strategies regarding PMS, and it may provide some practical suggestions on clinical decisions.

In this research, we found that PMS mostly occurred in young men with a median age of 31, which was in line with previous researches [5, 8, 13]. Patients under 40 years held a majority of the entire population, while the survival of which was superior to those over 40 years, and it was even poorer in those over 60 years. Thus, young age was associated with favorable survival, and similar findings had been proved in prior studies [13, 14]. Patients under 40 years had a higher 10-year OS rate than the older (100% versus 66%, *P*=0.013) [13]. Even though only 2.9% of the females were diagnosed with PMS, we found no distinction in survival concerning gender. Furthermore, black patients and married patients seem to perform well and obtain a long-term CSS, but with no statistical significance (black, HR = 0.42, *P*=0.067; married, HR = 0.57, *P*=0.064).

With slow growth and inconspicuous symptoms, seminoma was found to be of a large size when diagnosed [5]. In our analysis, the median tumor size was 11.6 cm, similar to Dechaphunkul's study [20], but much larger than the 5 cm reported before [5]. We found that the tumor size was not associated with either OS (*P*=0.821) or CSS (*P*=0.967). However, the extension of tumors at the primary site would significantly affect both OS and CSS, which was also illustrated in Liu's study [17].

Previous studies indicated that those with PMS were treated with radiotherapy and proved effective [21–23]. A retrospective study showed that nine of eleven patients with PMS were locally controlled after radiotherapy and proposed that PMS was radiosensitive, compared with nonseminomatous carcinoma [22]. Moreover, it was noted that they all received high-dose radiotherapy, with a median of at least 4,000 cGy [22, 23], whereas another retrospective study included 13 cases in 50 years and showed that those with surgery and postoperative radiotherapy might have a relapse in the lung or other distant sites, which were not in the radiation area [8]. Prior studies figured out that chemotherapy was effective in PMS, and several studies pointed out that radiotherapy may not be necessary [5, 20, 24]. In our research, 85.5% of patients received chemotherapy, while no distinction was noticed in survival regarding chemotherapy. However, multivariate analysis was conducted among patients over 40 years in the primary cohort, which showed improved survival for chemotherapy in both OS and CSS (*P* < 0.001). Cisplatin-based chemotherapy was recommended in the initial management, in contrast to the salvage therapy after radiotherapy when met with an unsatisfactory response [1, 20, 25]. Furthermore, the initial management of radiotherapy might lead to a higher relapse rate [1, 26]. Due to the SEER database's inherent limitation, disease-free survival (DFS) and progression-free (PFS) were not available for analysis. Adjuvant chemotherapy was considered to improve PFS, but not OS [27], and chemotherapy may improve DFS or PFS rather than radiotherapy [1, 5, 28].

In most cases, radical surgery played an essential role in patients without metastatic lesions [16, 20, 29, 30]. A definitive conclusion has not been concluded previously concerning the potential benefit of surgery as an initial treatment [31]. At the same time, we found that surgery was associated with improved OS and CSS in the training cohort. Nevertheless, the surgery modalities and surgery margin have not been investigated, just that we could not identify the surgery details in the SEER database. Thus, more aggressive procedures may be preferred to decrease the potential relapse and metastasis further. Systemic chemotherapy and salvage operation could be taken into consideration for unresectable cases [32]. A retrospective study focused on treatment strategies indicated that patients with three modalities of treatments (including chemotherapy, radiotherapy, and operation) would have poorer survival than those with two modalities of treatments. It might result from treatment-related toxicities, and besides, they proposed that a single modality of treatment was not sufficient to thoroughly remove the tumor cells [17].

We constructed nomograms to predict the survival, and the 1-, 5-, and 10-year OS and CSS rates could be calculated visually. *C*-index, calibration curve, and ROC curve were employed to identify the model's prediction power, which was evaluated by *C*-index and AUC quantitatively. Our prediction model showed a moderate prediction power in internal and external validation, for whose AUC values were all over 0.7. A prognostic factor-based staging system has been established by the International Germ-Cell Cancer Collaborative Group (IGCCCG) only for metastatic seminoma. In the research, the predominant evaluation indicator was the presence of nonpulmonary visceral metastases, and seminoma was then divided into two groups: good prognosis (5-year OS, 86%) and intermediate prognosis (72%) [10]. IGCCCG proposed another prognostic model of metastatic GCTs with first-line treatment failure in 2010, in which seminomas were only included in the validation cohort, and Harrell's C statistic was 0.661 and referred to a low prediction power [15]. The primary site of the tumor, the response of prior treatment, serum tumor biomarkers, and metastasis were included qualitatively in the prediction model, while it only focused on metastatic seminoma, and other demographics or tumor biological characteristics was not involved. Notably, our research cared about mediastinal seminoma specifically, and included all stages and more clinical characteristics.

There are several limitations in our study, including the inherent limitations of a retrospective study. For the sake of the seventh edition of TNM staging system which has been employed since 2010, the EOD staging system was adopted for analysis, which was comparable with the third edition of TNM staging. Furthermore, several potential prognostic factors were not available in the SEER database, including physical status, some details of the treatment, serum tumor biomarkers, and response to therapy. Thus, our findings were supposed to be interpreted carefully and called for more validations in multicenter studies.

## 5. Conclusion

PMS was uncommon and mainly occurred in young men, while those under 40 years would have better survival than those over 40 years. Surgery was the potential effective treatment rather than radiotherapy, and those over 40 years would benefit from chemotherapy. The prediction model with nomogram could effectively predict the long-term survival of PMS and help clinicians to make clinical decisions.

## Figures and Tables

**Table 1:** Clinical characteristics of patients with primary mediastinal seminoma in the primary cohort, training cohort, and validation cohort.

Variable	Primary cohort (*n* = 476)		Training cohort (*n* = 333)		Validation cohort (*n* = 143)		*P* value
	Number	%	Number	%	Number	%	
Sex							1.000
Male	462	97.1	323	97.0	139	97.2	
Female	14	2.9	10	3.0	4	2.8	

Age (years)							0.495
<20	47	9.9	30	9.0	17	11.9	
20∼40	299	62.8	209	62.8	90	62.9	
40∼60	112	23.5	79	23.7	33	23.1	
≥60	18	3.8	15	4.5	3	2.1	

Year of diagnosis							0.316
<2004	213	44.7	154	46.2	59	41.3	
≥2004	263	55.3	179	53.8	84	58.7	

Race							0.438
White	387	81.3	268	80.5	119	83.2	
Black	42	8.8	33	9.9	9	6.3	
Other*∗*	47	9.9	32	9.6	15	10.5	

Marital status							0.285
Single	245	51.5	164	49.2	81	56.6	
Married	188	39.5	136	40.8	52	36.4	
Other#	43	9.0	33	9.9	10	7.0	

Extent of disease							0.702
Localized	155	32.6	107	32.1	48	33.6	
Regional	134	28.1	99	29.7	35	24.5	
Distant	131	27.5	89	26.7	42	29.4	
Unknown	56	11.8	38	11.4	18	12.6	

Extent of primary site							0.986
CSO	101	21.2	70	21.0	31	21.7	
LNOS	92	19.3	64	19.2	28	19.6	
ACT	28	5.9	20	6.0	8	5.6	
AO/S	106	22.3	74	22.2	32	22.4	
FCE	22	4.6	17	5.1	5	3.5	
Unknown	127	26.7	88	26.4	39	27.3	

Lymph nodes involved*∗*							0.606
Yes	107	22.5	79	23.7	28	19.6	
No	268	56.3	185	55.6	83	58.0	
Unknown	101	21.2	69	20.7	32	22.4	

Metastasis							0.770
Yes	131	27.5	89	26.7	42	29.4	
No	288	60.5	205	61.6	83	58.0	
Unknown	57	12.0	39	11.7	18	12.6	

Size of tumor (cm)							0.383
<10	101	21.2	65	19.5	36	25.2	
≥10	201	42.2	144	43.2	57	39.9	
Unknown	174	36.6	124	37.2	50	35.0	

Surgery							0.036
Performed	113	23.7	88	26.4	25	17.5	
NP/NA	363	76.3	245	73.6	118	82.5	

Radiation							0.574
Performed†	94	19.7	68	20.4	26	18.2	
NP/NA	382	80.3	265	79.6	117	81.8	

Chemotherapy							0.836
Performed	407	85.5	284	85.3	123	86.0	
NP/NA	69	14.5	49	14.7	20	14.0	

CSO, confined to site of origin; LNOS, localized (not of specified); ACT, extend to adjacent connective tissue; AO/S, adjacent organs/structures; FCE, further contiguous extension; NP/NA, not performed or unknown. *∗*: Asian or Pacific Islander, American Indian/Alaska native, unknown. #: divorced, separated, widowed, and unknown. †: beam radiation and radiation (not otherwise specified method or source not specified).

**Table 2:** Univariate analysis and multivariate analysis of overall survival and cancer-specific survival in the training cohort.

Variable	Overall survival			Cancer-specific survival		
	Univariate analysis	Multivariate analysis		Univariate analysis	Multivariate analysis	
	*P* value	HR (95%CI)	*P* value	*P* value	HR (95%CI)	*P* value
Sex	0.004			0.002		
Male		Ref.			Ref.	
Female		1.43 (0.57–3.61)	0.445		1.55 (0.55–4.32)	0.404

Age (years)	<0.001			<0.001		
<20		Ref.			Ref.	
20∼40		1.56 (0.70–3.51)	0.279		1.67 (0.65–4.29)	0.284
40∼60		2.64 (1.09–6.41)	0.032		2.67 (0.94–7.58)	0.065
≥60		5.26 (1.75–15.81)	0.003		7.93 (2.22–28.32)	0.001

Year of diagnosis	0.036			0.370		
<2004		Ref.			Ref.	
≥2004		0.70 (0.44–1.11)	0.131		1.00 (0.59–1.69)	0.989

Race	0.291			0.967		
White		Ref.			Ref.	
Black		0.77 (0.38–1.54)	0.461		0.42 (0.16–1.06)	0.067
Other		0.89 (0.42–1.90)	0.760		1.01 (0.44–2.35)	0.974

Marital status	0.075			0.149		
Single		Ref.			Ref.	
Married		0.74 (0.46–1.22)	0.239		0.57 (0.32–1.03)	0.064
Other		1.29 (0.68–2.47)	0.438		1.11 (0.51–2.40)	0.789

Extent of primary site	<0.001			<0.001		
CSO		Ref.			Ref.	
LNOS		1.14 (0.53–2.42)	0.736		1.03 (0.40–2.65)	0.944
ACT		2.55 (0.94–6.87)	0.065		2.54 (0.76–8.51)	0.130
AO/S		1.86 (0.93–3.74)	0.080		1.79 (0.77–4.14)	0.173
FCE		1.75 (0.59–5.21)	0.313		2.87 (0.86–9.58)	0.087
Unknown		2.56 (1.07–6.13)	0.034		3.36 (1.21–9.31)	0.020

Lymph nodes involved	<0.001			<0.001		
No		Ref.			Ref.	
Yes		1.16 (0.68–1.99)	0.585		1.68 (0.90–3.15)	0.105
Unknown		1.02 (0.54–1.92)	0.946		1.48 (0.70–3.15)	0.304

Metastasis	<0.001			<0.001		
No		Ref.			Ref.	
Yes		2.05 (1.12–3.75)	0.020		1.88 (0.94–3.75)	0.073
Unknown		0.34 (0.13–0.93)	0.035		0.14 (0.03–0.56)	0.006

Tumor size (cm)	0.246			0.202		
<10		Ref.			Ref.	
≥10		1.07 (0.61–1.88)	0.821		1.01 (0.52–1.98)	0.967
Unknown		0.98 (0.56–1.74)	0.955		1.02 (0.53–1.96)	0.955

Surgery	0.012			0.029		
Performed		Ref.			Ref.	
NP/NA		1.86 (1.14–3.03)	0.013		1.84 (1.02–3.31)	0.041

Radiation	0.339			0.677		
Performed		Ref.			Ref.	
NP/NA		1.11 (0.70–1.74)	0.664		1.28 (0.74–2.23)	0.375

Chemotherapy	0.383			0.679		
Performed		Ref.			Ref.	
NP/NA		1.56 (0.90–2.68)	0.112		1.70 (0.86–3.38)	0.128

HR, hazard ratio; CI, confidence interval; Ref., reference; CSO, confined to site of origin; LNOS, localized (not otherwise specified); ACT, extend to adjacent connective tissue; AO/S, adjacent organs/structures; FCE, further contiguous extension; NP/NA, not performed or unknown.

## Data Availability

In the study, the authors obtained the data from the SEER database, which was open access for research purposes. The authors were permitted to access the data at the website (http://www.seer.cancer.gov) with the identifier, 17219-Nov 2018. For the sake of the data anonymization in the SEER database and elimination of patient identification, the research ethics approval was not required.

## References

[1] Fizazi K., Culine S., Droz J.-P. (1998). Initial management of primary mediastinal seminoma: radiotherapy or cisplatin-based chemotherapy?. *European Journal of Cancer*.

[2] Woolner L. B., Jamplis R. W., Kirklin J. W. (1955). Seminoma (germinoma) apparently primary in the anterior mediastinum. *New England Journal of Medicine*.

[3] Lee H., Lim J.-K., Lee S. Y., Shin K. M. (2018). Granulomatous reaction of primary mediastinal seminoma leading to diagnostic delay: a case report. *Journal of Thoracic Disease*.

[4] Clamon G. H. (1983). Management of primary mediastinal seminoma. *Chest*.

[5] Bokemeyer C., Nichols C. R., Droz J.-P. (2002). Extragonadal germ cell tumors of the mediastinum and retroperitoneum: results from an international analysis. *Journal of Clinical Oncology*.

[6] Hartmann J. T., Nichols C. R., Droz J.-P. (2002). Prognostic variables for response and outcome in patients with extragonadal germ-cell tumors. *Annals of Oncology*.

[7] Pauniaho S.-L., Salonen J., Helminen M., Vettenranta K., Heikinheimo M., Heikinheimo O. (2012). The incidences of malignant gonadal and extragonadal germ cell tumors in males and females: a population-based study covering over 40 years in Finland. *Cancer Causes & Control*.

[8] Takeda S.-i., Miyoshi S., Ohta M., Minami M., Masaoka A., Matsuda H. (2003). Primary germ cell tumors in the mediastinum. *Cancer*.

[9] Rusner C., Trabert B., Katalinic A., Kieschke J., Emrich K., Stang A. (2013). Incidence patterns and trends of malignant gonadal and extragonadal germ cell tumors in Germany, 1998–2008. *Cancer Epidemiology*.

[10] (1997). International germ cell consensus classification: a prognostic factor-based staging system for metastatic germ cell cancers. International germ cell cancer collaborative group. *Journal of Clinical Oncology*.

[11] Oing C., Oechsle K., Necchi A. (2017). Impact of primary metastatic bone disease in germ cell tumors: results of an international global germ cell tumor collaborative group G3 registry study. *Annals of Oncology*.

[12] Kier M. G., Lauritsen J., Mortensen M. S. (2017). Prognostic factors and treatment results after bleomycin, etoposide, and cisplatin in germ cell cancer: a population-based study. *European Urology*.

[13] Napieralska A., Majewski W., Osewski W., Miszczyk L. (2018). Primary mediastinal seminoma. *Journal of Thoracic Disease*.

[14] Moran C. A., Suster S., Przygodzki R. M., Koss M. N. (1997). Primary germ cell tumors of the mediastinum. *Cancer*.

[15] Lorch A., Beyer J., Bascoul-Mollevi C. (2010). Prognostic factors in patients with metastatic germ cell tumors who experienced treatment failure with cisplatin-based first-line chemotherapy. *Journal of Clinical Oncology*.

[16] Rivera C., Arame A., Jougon J. (2010). Prognostic factors in patients with primary mediastinal germ cell tumors, a surgical multicenter retrospective study. *Interactive CardioVascular and Thoracic Surgery*.

[17] Liu T. Z., Zhang D. S., Liang Y. (2011). Treatment strategies and prognostic factors of patients with primary germ cell tumors in the mediastinum. *Journal of Cancer Research and Clinical Oncology*.

[18] Cronin K. A., Ries L. A. G., Edwards B. K. (2014). Preface. *Cancer*.

[19] Weiss A., Chavez-MacGregor M., Lichtensztajn D. Y. (2018). Validation study of the American Joint committee on cancer eighth edition prognostic stage compared with the anatomic stage in breast cancer. *JAMA Oncology*.

[20] Dechaphunkul A., Sakdejayont S., Sathitruangsak C., Sunpaweravong P. (2016). Clinical characteristics and treatment outcomes of patients with primary mediastinal germ cell tumors: 10-years’ experience at a single institution with a bleomycin-containing regimen. *Oncology Research and Treatment*.

[21] Economou J. S., Trump D. L., Carmack Holmes E., Eggleston J. E. (1982). Management of primary germ cell tumors of the mediastinum. *The Journal of Thoracic and Cardiovascular Surgery*.

[22] Kersh C. R., Constable W. C., Hahn S. S (1990). Primary malignant extragonadal germ cell tumors. An analysis of the effect of the effect of radiotherapy. *Cancer*.

[23] Osada H., Kojima K., Yamate N. (1998). Primary mediastinal seminoma. *The Japanese Journal of Thoracic and Cardiovascular Surgery*.

[24] Logothetis C. J., Samuels M. L., Selig D. E. (1985). Chemotherapy of extragonadal germ cell tumors. *Journal of Clinical Oncology*.

[25] Jain K. K., Bosl G. J., Bains M. S., Whitmore W. F., Golbey R. B. (1984). The treatment of extragonadal seminoma. *Journal of Clinical Oncology*.

[26] Bokemeyer C., Droz J.-P., Horwich A. (2001). Extragonadal seminoma. *Cancer*.

[27] Fizazi K., Tjulandin S., Salvioni R. (2001). Viable malignant cells after primary chemotherapy for disseminated nonseminomatous germ cell tumors: prognostic factors and role of postsurgery chemotherapy-results from an international study group. *Journal of Clinical Oncology*.

[28] Wang J. L., Yu H., Guo Y (2012). A single institution, retrospective study of treatment experience in primary mediastinal germ cell tumors: elucidating the significance of systemic chemotherapy. *Chinese Medical Journal*.

[29] Gerl A., Clemm C., Lamerz R., Wilmanns W. (1996). Cisplatin-based chemotherapy of primary extragonadal germ cell tumors: a single institution experience. *Cancer*.

[30] Kuwano H., Tsuchiya T., Murayama T. (2014). Outcomes of combined modality therapy for patients with stage III or IV mediastinal malignant germ cell tumors. *Surgery Today*.

[31] Rosti G., Secondino S., Necchi A., Fornarini G., Pedrazzoli P. (2019). Primary mediastinal germ cell tumors. *Seminars in Oncology*.

[32] Liu Y., Wang Z., Peng Z. M. (2014). Management of the primary malignant mediastinal germ cell tumors: experience with 54 patients. *Diagnostic Pathology*.

